# SGMLN: Sentiment-Guided Mutual Learning Network for Multimodal Sarcasm Detection

**DOI:** 10.3390/s26082304

**Published:** 2026-04-08

**Authors:** Yiran Wang, Xin Zhao, Yongtang Bao

**Affiliations:** College of Computer Science and Engineering, Shandong University of Science and Technology, Qingdao 266590, China; wangyr@sdust.edu.cn (Y.W.); 202283060010@sdust.edu.cn (X.Z.)

**Keywords:** multimodal sarcasm detection, sentiment-guided, mutual learning, multimodal fusion

## Abstract

Social networks such as Twitter have grown rapidly and are now flooded with sarcastic comments, both in text and in images. Detecting sarcasm in multimodal data has significant social value and is attracting increasing research attention. However, most studies overlook the role of sentiment, even though sentiment information in text is closely linked to clues of sarcasm. Additionally, few consider how text and images align semantically. To address these issues, we propose a sentiment-guided mutual learning network (SGMLN) for multimodal sarcasm detection. SGMLN utilizes sentiment information to inform the combination of text and image features, and employs mutual learning to facilitate knowledge sharing among classifiers. We design a sentiment-guided attention layer that injects sentiment into both modalities, producing features that capture sarcasm more effectively. Sentic-BERT extracts sentiment-aware vectors from text, using word-level sentiment as a mask. In mutual learning, a logistic distribution function measures differences between classifiers, improving knowledge transfer between modalities. This step boosts multimodal understanding and model performance. By introducing sentiment-aware representations and semantic alignment, SGMLN bridges the gap between text and images, making them more consistent. Experiments on public datasets demonstrate that our model is effective and outperforms alternatives.

## 1. Introduction

Sarcasm occurs when a speaker’s intended meaning contradicts their literal words; this form of expression is now commonplace on social media and e-commerce platforms. As online communication evolves, users increasingly leave sarcastic comments. As a result, detecting sarcasm is crucial for effective sentiment analysis [1], question-answering [2], and opinion mining [3]. By identifying sarcastic remarks, platforms can infer user preferences, improve service quality, increase user satisfaction, and deliver social value [4,5].

Initially, sarcasm detection focused on textual content. Kamal et al. [6] were among the first to apply deep learning approaches to this task, proposing a model based on Long Short-Term Memory (LSTM), Bidirectional Gated Recurrent Units (BiGRUs), and an attention layer, which achieved promising results. Building on this direction, Long et al. [7] introduced a model that leverages Bidirectional Long Short-Term Memory (Bi-LSTM) and Multi-Head Attention (MHAT). In their approach, the Bi-LSTM addressed long-term dependencies by capturing contextual information, while the MHAT was adept at identifying key parts of sentences.

**Figure 1:**
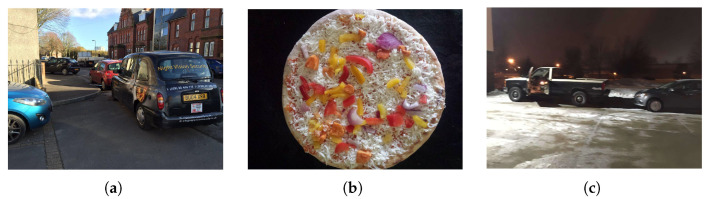
Examples of multimodal sarcastic data. (**a**) great parking by one of your guys! (**b**) cheers for the sweet chills chicken pizza, overwhelmed with the amount of chicken. (**c**) thanks for leaving me room to back out.

User participation in discussions about hot topics on social networking platforms such as Facebook is increasing, and these discussions are predominantly multimodal, involving images, text, and audio [8,9]. This shift presents new challenges for sarcasm detection. As illustrated in Figure 1, multimodal data—compared to unimodal data that relies solely on text or images—can provide a more comprehensive, accurate, and diverse perspective. As a result, an increasing number of researchers are focusing on detecting multimodal sarcasm. For example, some previous work has fused multimodal features by directly concatenating text features from text encoders and image features from image encoders.

With the advent of attention mechanisms [10,11], researchers have increasingly leveraged attention structures to extract and compare features across modalities. These attention-based methods have been instrumental in uncovering inconsistencies between modalities after feature extraction. However, Liu et al. [12] observed that earlier research mostly addresses fine-grained inconsistencies within textual and visual content while overlooking more complex cross-modal interactions. The importance of such interactions has been highlighted, particularly in tasks like cross-modal retrieval [13] and image–text matching [14,15]. Building on this, Wen et al. [16] identified a semantic link between cross-modal sarcasm and attitudes in text, emphasizing the need for models that handle such semantic interplay. Furthermore, the growing maturity of graph neural networks [17,18] has enabled researchers to better model intricate inter-modal relationships and facilitate the detection of modality inconsistencies. In addition to these advancements, incorporating common-sense knowledge has also proven valuable, prompting its integration into affective computing, specifically in sarcasm detection [19,20]. For example, Yue et al. [21] employed the ConceptNet knowledge base and cross-modal semantic-similarity measures to guide image–text feature fusion. In sum, recent work emphasizes the need to move beyond low-level inconsistencies and develop models that integrate complex, knowledge-driven, cross-modal cues.

Although these methods can effectively identify implicit sarcasm across modalities and improve sarcasm detection to some extent, they do not fully account for the critical role of sentiment. They also overlook the semantic gap. The semantic gap refers to the challenge of aligning and collaborating between text and image features on the semantic level. This is especially true when their dimensions and semantics differ significantly. Such differences hinder the capture of cross-modal inconsistencies. Therefore, we propose a sentiment-guided multimodal mutual learning model (SGMLN) for sarcasm detection. We use the sentimental information of each word in the text sentence as a mask for Sentic-BERT. This approach helps extract sentiment-aware vectors that capture sentimental clues within the text. In the initial fusion phase, we utilize sentiment-aware vectors as query vectors for the sentiment-guided fusion attention layer. This attention layer infuses sentimental information into the text and image features. To transfer sarcastic information between the two modalities, we implement mutual learning between the image and text network classifiers. We use a logistic distribution to assess the classifier’s divergence. This enables cross-modal knowledge transfer and reduces the semantic gap between image and text features. By integrating the learning processes of both classifiers, we achieve collaborative enhancement of sarcasm detection.

The main contributions of this study are summarized as follows:

(1) We introduce the SGMLN model for sarcasm detection. This practical tool integrates the interdependence between image and text modalities by leveraging sentiment information. By leveraging mutual learning between two classifiers, we increase semantic consistency between textual and visual streams. This approach improves sarcasm detection. Our experiments use a widely adopted multimodal sarcasm detection benchmark. These tests yield outstanding performance and show the model’s potential for real-world use.

(2) In the initial fusion phase, we propose replacing the Sentic-BERT mask with the sentimental information degree of each word to extract sentiment-aware vectors. These vectors serve as queries for the sentiment-guided fusion attention layer, enabling the infusion of sentiment into image and text features to refine and integrate them.

(3) During the mutual learning phase, the image and text network classifiers exchange knowledge. We use Kullback–Leibler (KL) divergence to minimize differences between the classifiers and bridge the semantic disparity between visual and textual representations.

## 2. Related Work

### 2.1. Multimodal Sarcasm Detection

The core challenge in this field lies in effectively extracting and integrating heterogeneous features from both text and images to identify semantic incongruity. Recent research has evolved from simple feature concatenation to sophisticated alignment strategies.

#### 2.1.1. Early Frameworks and Benchmark Construction

Initial research focused on establishing the foundational infrastructure for multimodal analysis. Schifanella et al. [22] were the first to introduce multimodality into sarcasm detection, proposing two model frameworks for extracting sarcastic features from textual and visual modalities. A landmark contribution was made by Cai et al. [23], who constructed the widely-used Twitter dataset and proposed a Hierarchical Fusion Model (HFM). By utilizing image attributes to guide textual feature fusion, their work established the basic paradigm of “cross-modal interaction”. While foundational, these early models relied primarily on coarse-grained global features, leaving room for more fine-grained semantic alignment.

#### 2.1.2. Inconsistency Modeling via Attention Mechanisms

Subsequent studies shifted toward capturing subtle contradictions through attention-based alignment. Pan et al. [24] further refined the model proposed by Cai et al. [23] by focusing on inconsistencies across images, text, and labels. They built a co-attention matrix to represent the association between textual content and labels, employing BERT for textual representation and ResNet-152 to extract visual features. However, co-attention primarily focuses on surface-level alignment between modalities and may fail to capture subtle sentiment contradictions or deep semantic inconsistencies, which are crucial for understanding sarcasm. After comparing various methods, they found that an integrated network combining a Transformer and image encoders significantly improved multimodal sarcasm detection performance. Similarly, Tian et al. [25] explored the impact of modal input sequences on detection performance by introducing a modality-order-driven attention module. These works demonstrate the immense potential of Transformers in boosting performance; however, as noted in our analysis, they often focus on surface-level alignment and may overlook deeper emotional contradictions.

#### 2.1.3. Knowledge Enhancement and Complex Relationship Mining

Recent advancements have sought to incorporate higher-dimensional signals. Lu et al. [26] proposed a fact–emotion inconsistency combination network that reveals multimodal sarcastic relationships by exploring the factual differences and emotional incongruities in multimodal information. Bao et al. [27] presented HIAN, a hybrid interactive attention network for multimodal sarcasm detection that integrates class words, text, and images. Using bidirectional LSTMs and interactive attention, the model achieved relatively good performance on sarcasm detection tasks. Wang et al. [28] introduced a relational context learning and multiplex fusion network that models text–image interactions and fuses multimodal features, leading to relatively advanced results in sarcasm detection. Yu et al. [29] developed a cross-modal interaction and multi-view misalignment framework that employs co-attention, structured captions, and sentiment-aware misalignment modeling to better capture sarcastic cues, demonstrating promising effectiveness across various benchmarks. These approaches highlight that sarcasm is not merely a data-matching problem but a cognitive one, directly inspiring the sentiment-prioritized design of our SGMLN.

### 2.2. Mutual Learning and Sentiment Guidance

This section reviews strategies for knowledge transfer and the utilization of auxiliary affective information to bridge the semantic gap.

#### 2.2.1. Deep Mutual Learning and Cross-Modal Synergy

Instead of conventional teacher–student distillation paradigms, recent research has increasingly explored collaborative optimization strategies that allow multiple networks to learn jointly during training. Reference [30] proposed a multimodal fusion model based on mutual learning. This model facilitates mutual learning between the image and text modalities regarding their feature representations. As a result, image features acquire rich semantic information from text features, and text features can enhance their understanding of semantics from image features, effectively improving the accuracy of multimodal classification tasks. Building upon this idea, Qiao et al. [31] incorporated mutual learning into local semantic guidance frameworks to enhance fine-grained feature alignment, while Wang et al. [32] extended the paradigm to structural alignment tasks, demonstrating its effectiveness in reducing discrepancies between heterogeneous modalities.

These studies collectively suggest that collaborative learning mechanisms can promote more balanced representation learning by encouraging consistency across modality-specific classifiers. However, existing approaches typically rely on direct prediction alignment, which may not fully address distributional differences between visual and textual feature spaces. Motivated by these observations, the proposed framework adopts a mutual learning strategy to facilitate smoother cross-modal knowledge transfer.

#### 2.2.2. Affective Signal-Guided Sarcasm Analysis

The role of affective information in sarcasm detection has attracted growing attention, as sarcasm frequently arises from incongruity between expressed sentiment and contextual meaning. Chauhan et al. [33] demonstrated that incorporating emotional cues within a multi-task learning framework can improve sarcasm recognition by providing auxiliary supervision signals. Subsequent studies further explored sentiment-aware modeling from different perspectives. Ye et al. [34] investigated sentiment-aware pre-training strategies to enhance contextual representation learning, whereas Ou and Li [35] (DCMG) employed graph convolutional networks to explicitly model inconsistencies between semantic content and emotional polarity.

Taken together, these works indicate that sentiment information can serve not merely as an auxiliary feature but as an important signal for identifying implicit pragmatic meaning. Nevertheless, many existing approaches incorporate affective cues only at the feature level, without explicitly guiding cross-modal interaction. Inspired by these limitations, the present study integrates external affective knowledge to support sentiment-aware fusion across modalities.

#### 2.2.3. Sarcasm Detection in the Multimodal Large Language Model Era

With the rapid development of foundation models, recent studies have begun to evaluate the capability of Multimodal Large Language Models (MLLMs) in complex cognitive tasks such as sarcasm detection. Wang et al. [36] conducted a large-scale benchmark using state-of-the-art models, including GPT-4o, and showed that, despite strong general knowledge, MLLMs often struggle with fine-grained affective incongruity and subtle semantic–visual inconsistencies, particularly in zero-shot settings where predictions tend to rely excessively on textual signals. To improve reasoning performance, Zhang et al. [37] proposed Commander-GPT, a modular multi-agent framework that decomposes sarcasm understanding into specialized subtasks and integrates intermediate outputs for final prediction. Although this design enhances reasoning capability and interpretability, it introduces substantial computational cost and inference latency due to repeated LLM interactions.

In contrast, task-specific architectures remain an important direction for efficient deployment. The proposed SGMLN complements existing MLLM-based approaches by emphasizing explicit sentiment-guided interaction and mutual learning mechanisms. Rather than relying on large-scale reasoning, SGMLN focuses on affective alignment across modalities, achieving competitive performance with significantly lower computational overhead.

## 3. Methodology

**Figure 2:**
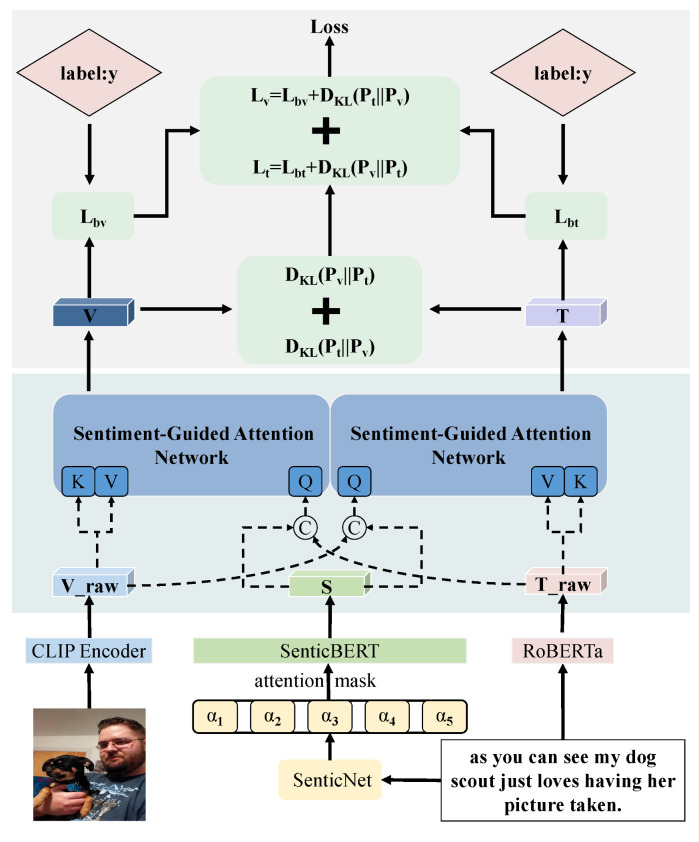
Overall architecture of the proposed SGMLN model.

This section introduces our proposed SGMLN model for multimodal sarcasm detection. As shown in Figure 2, the SGMLN framework leverages emotional information and mutual learning to align and fuse image and text features. The workflow begins with Sentic-BERT extracting emotional text features to support interaction with image features. Following this, the RoBERTa and Vision Transformer (ViT) models extract general textual and image features, respectively. An emotion-guided attention layer is then applied to facilitate interaction among sentimental, textual, and image features. Finally, a mutual learning strategy enables information transfer between image and text classifiers, thereby reducing the semantic gap between the modalities.

### 3.1. Task Definition

The task of multimodal sarcasm detection can be formulated as follows: Given an image–text pair (*I*,*T*), where each pair consists of a sentence *T* containing *n* words and an image *I* related to the text, the objective of our model is to learn a classifier that accurately predicts the sarcasm label for the given image–text pair.

### 3.2. Sentimental Feature Encoding Module

SenticNet is a knowledge base encompassing a semantic, affective, and polarity-linked set of 100,000 natural language concepts. As a framework, it integrates a suite of sentiment analysis tools and techniques that amalgamate common-sense reasoning, psychology, linguistics, and machine learning. Leveraging artificial intelligence and natural language processing, SenticNet serves as a sentiment analysis framework that identifies and interprets emotional tendencies in textual data.

**Figure 3:**
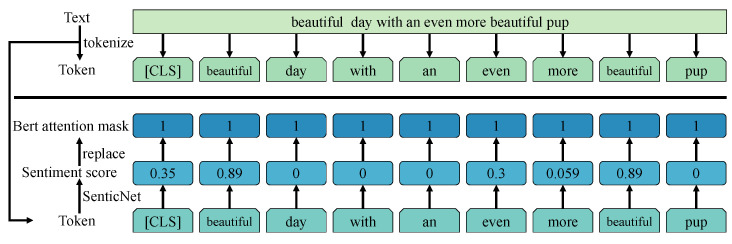
The attention mask of Sentic-BERT.

As depicted in Figure 3, we retrieve the affective polarity scores $\alpha_{n}$ of each word in the text from the SenticNet lexicon. We substitute the attention masks of each word in the pre-trained BERT, which is trained on a large-scale dataset, with the affective polarity scores of the respective words:

(1) $$\alpha_{i}=\mathrm{SenticNet}\left(a_{i}\right),$$

where $a_{i}$ represents each word that constitutes the text, i∈[1,n], and *n* is the number of words.

Employing Sentic-BERT as a sentiment vector encoder, we derive sentiment-guided vector *S* from the text:

(2) S=SenticBERT(text).

To better capture the nature of multimodal sarcasm, the masking strategy is designed not as an information reduction process but as a training mechanism that discourages excessive reliance on explicit sentiment expressions. During training, selectively masking strongly sentiment-bearing words encourages the model to infer sarcasm from the remaining contextual semantics and their interaction with visual features. This allows neutral or literal textual content to remain informative while supporting the modeling of cross-modal incongruity, a key characteristic of sarcastic expressions. Empirical results further indicate that this strategy improves model robustness when explicit sentiment cues are limited.

### 3.3. Image and Text Encoding Module

The RoBERTa model has been pre-trained on a vast corpus of data. As a result, it has acquired a rich understanding of language and semantic representation. This provides it with strong generalization capabilities, enabling a deeper and more precise comprehension of the input text. The CLIP model, pre-trained on numerous image–text pairs, excels at linking and understanding information from both text and images. It captures high-level semantic details in images and excels at tasks involving image–text interaction. We use RoBERTa as a text encoder to extract general textual features. We also use the CLIP image encoder to extract image features. This lets us obtain features for model training that are agnostic to specific domains.

Initially, the text is tokenized into a sequence of tokens $\mathrm{Text}=\left\{\left[CLS\right],t_{1},t_{2},...,t_{n-1}\right\}$, where [CLS] represents the global token, and the length of the text sentence is denoted by the number of tokens. This token sequence is then fed into RoBERTa, which produces contextualized feature representations for each token in the sequence:

(3) $$T_{raw}=\mathrm{RoBERTa}\left(\mathrm{Text}\right)=\left[t_{1},t_{2},...,t_{n}\right],$$

where $T_{raw}\in R^{n\times d_{t}}$, $t_{n}$ is the embedding feature of each token sequence of the text. $d_{t}$ is the embedding dimension of the text.

For image data, the input image *I* is divided into *m* patches, and the image is then passed through the pre-trained image encoder (ViT) from CLIP to obtain visual features. The process is as follows:

(4) $$V_{raw}=\mathrm{ViT}\left(I\right)=\left[v_{1},v_{2},...,v_{m}\right],$$

where $V_{raw}\in R^{m\times d_{v}}$, $v_{m}$ is the embedding feature of each patch of the image, and $d_{v}$ is the embedding dimension of the image.

### 3.4. Sentiment-Guided Attention Module

Sarcasm implicitly conveys genuine emotions through positive expressions, like dissatisfaction and resentment. For instance, in the sentence “great parking by one of your guys” in Figure 1a, the word “great” expresses a positive emotion. Meanwhile, the crowded space in the picture represents a negative emotion, creating a strong contradiction. Thus, this example is sarcastic. Detecting emotions in sarcastic texts and re-applying these cues to images and texts can aid multimodal sarcasm detection. We designed a sentiment-guided attention module to incorporate sentiment from sarcastic texts into images and text.

For each modality *r*$\left(r\in \left\{v,t\right\}\right)$, we concatenate the sentiment embedding *S* with the complementary modality embedding *x*$\left(x\in \left\{V_{raw},T_{raw}\right\}\right)$. We then map them to the query matrix Query(Q) of the sentimental attention fusion layer. The original features *y*$\left(y\in \left\{V_{raw},T_{raw}\right\}\right)$ are mapped to the key value matrix Key(K) and Value(V). The sentimental attention fusion layer then integrates the semantic information between modalities and the sentimental cues in *S* into the original features *y*. This yields the preceptive sentiment features $X_{r}^{i}$. The process can be expressed as:

(5) $$\begin{array}{cc} Q_{r}^{i} & =W_{r}^{Q}\cdot \left[S,x\right]+b_{r}^{Q},\\ K_{r}^{i} & =W_{r}^{K}\cdot \left[S,x\right]+b_{r}^{K},\\ V_{r}^{i} & =W_{r}^{V}\cdot \left[S,x\right]+b_{r}^{V},\\ y_{r}^{i} & =\mathrm{softmax}\left(\tanh\left(K_{r}^{i}+Q_{r}^{i}\right)\right)\cdot V_{r}^{i},\\ X_{r}^{i} & =W_{m}\cdot \left[S,x\right]+\mathrm{proj}\left(y_{r}^{i}\right), \end{array}$$

where $W_{r}^{Q}$, $W_{r}^{K}$, $W_{r}^{V}$, $W_{m}$, $b_{r}^{Q}$, $b_{r}^{K}$ and $b_{r}^{V}$ are the trainable parameters. proj is a mapping layer, and softmax is used to calculate the sentimental attention score of the original features, representing the degree of attention of the original features to sentiment.

### 3.5. Mutual Learning Module

First, the sentiment-guided image fusion module and the sentiment-guided text fusion module are used to capture intra-modal and inter-modal inconsistencies. Building on the recognition of potential consistency between these two modules, we next adopt a mutual learning strategy to enable knowledge sharing between the image and text modalities. In this approach, after sentiment perception, we feed each modality’s features $X_{r}^{i}$ into their respective fully connected layers. This process sequentially produces the predicted distribution $p_{v}$ for the image and $p_{t}$ for the text as follows:

(6) $$\begin{array}{cc} p_{v} & =\mathrm{softmax}(W_{v}\cdot X_{v}^{i}+b_{v}),\\ p_{t} & =\mathrm{softmax}(W_{t}\cdot X_{t}^{i}+b_{t}), \end{array}$$

where $W_{v}$, $W_{t}$, $b_{v}$ and $b_{t}$ are the trainable parameters.

We use the KL divergence [38] to quantify the discrepancy and encourage consistency between the two learning modules. Unlike prior knowledge transfer methods that ignore a sample’s prior knowledge, our approach transfers only reliable knowledge, preventing the propagation of errors. We introduce an indicator to control which sample predictions are transmitted, reducing the risk of incorrect outputs and mitigating the effect of modality noise in training. The knowledge transfer function is defined as:

(7) $$\begin{array}{c} L_{v\to t}=\mu_{1}D_{KL}(p_{v}\left|\right|p_{t}),\\ L_{t\to v}=\mu_{2}D_{KL}(p_{t}\left|\right|p_{v}), \end{array}$$

where (v→t) represents the transfer of knowledge from the image modality to the text modality; similarly, (t→v) represents the transfer of knowledge from the text modality to the image modality. $\mu_{i}\left(i\in \left\{1,2\right\}\right)$ are control parameters used to avoid incorrect knowledge transfer, and their definitions are as follows:

(8) $$\mu_{i}=\left\{\begin{array}{cc} 1 & \mathrm{if}\;\mathrm{argmax}(p_{v/t})=Y,\\ 0 & \mathrm{if}\;\mathrm{argmax}(p_{v/t})\neq Y, \end{array}\right.$$

where argmax represents the operation of obtaining the predicted label from the predicted result $p_{v/t}$, and $p_{v/t}$ is the predicted probability distribution. In this context, *Y* denotes the label.

Furthermore, the KL Losses $D_{KL}(p_{v}\left|\right|p_{t})$ and $D_{KL}(p_{t}\left|\right|p_{v})$ are defined as follows:

(9) $$\begin{array}{cc} D_{KL}(p_{v}\left|\right|p_{t}) & =\sum p_{v}\log\left(\frac{p_{v}}{p_{t}}\right),\\ D_{KL}(p_{t}\left|\right|p_{v}) & =\sum p_{t}\log\left(\frac{p_{t}}{p_{v}}\right). \end{array}$$

### 3.6. Optimization Objectives

For the SGMLN model, the training objective is to optimize all parameters by minimizing the overall loss function. The combined loss is formulated as follows:

(10) $$\begin{array}{cc} L^{v} & =L_{bce}^{v}+L_{t\to v},\\ L^{t} & =L_{bce}^{t}+L_{v\to t}, \end{array}$$

where $L_{bce}^{t}$ and $L_{bce}^{v}$ are binary cross-entropy losses of the text model and vision model. The definition of binary cross entropy loss $L_{bce}$ is as follows:

(11) $$\begin{array}{c} L_{bce}=-\frac{1}{N}\sum_{i=1}^{N}\left[y_{i}\log\left(p_{i}\right)+\left(1-y_{i}\right)\log\left(1-p_{i}\right)\right], \end{array}$$

where *N* is the number of samples, $y_{i}$ is the true label, and $p_{i}$ is the predicted probability of the positive class.

## 4. Experiments

### 4.1. Dataset

The proposed model is evaluated using a publicly available English-language dataset for multimodal sarcasm detection, as introduced by Cai et al. [23]. The dataset was selected based on its suitability for multimodal sarcasm analysis. Compared with text-only sarcasm corpora, MMSD provides naturally paired text–image samples collected from real-world social media posts, enabling the study of cross-modal incongruity, which is a key characteristic of sarcastic expression. The annotations were refined through manual verification following hashtag-based collection, improving label reliability beyond simple keyword supervision. With its relatively large scale and balanced number of sarcastic and non-sarcastic samples, the dataset offers sufficient diversity for evaluating multimodal fusion models and has therefore become a widely adopted benchmark in this research area. To improve detection accuracy, the dataset is preprocessed by removing explicit sarcasm indicators, such as “sarcasm”, “jokes”, and “irony”. Each instance comprises both textual and visual content. The dataset is subsequently partitioned into training, validation, and test sets in proportions of 80%, 10%, and 10%, respectively. Comprehensive dataset statistics are presented in Table 1.

Training complex models on limited datasets often leads to overfitting. In such cases, strong training performance fails to generalize to unseen data. Access to extensive datasets could alleviate this issue, but such conditions are rarely feasible. To address this gap, data augmentation is employed. It synthetically increases the volume and variety of training samples, thereby enhancing model robustness and reducing overfitting. In this context, a multimodal augmentation method, adapted from MixGen, is applied to boost model performance [39]. Drawing on task-specific analysis of sarcasm detection, we introduce a strategy that embeds an image $I_{insert}$ into a base image $I_{base}$ to generate a composite image $I_{new}$. A segment of text $T_{insert}$ is randomly placed within another text $T_{base}$ to form a new text $T_{new}$. By combining these techniques, our approach addresses diminished cross-modal consistency, a problem often caused by text-only augmentation in sarcasm detection. The detailed methodology is described below:

(12) $$\begin{array}{cc} \; & I_{new}=\varphi \cdot I_{base}+\left(1-\varphi \right)\cdot I_{insert},\\ \; & T_{new}=RandomInsert\left(T_{insert},T_{base},\varphi \right), \end{array}$$

where φ is the proportion retained. In this task, we set φ ≥ 0.7 to ensure semantic relationships match.

### 4.2. Implementation Details

This section presents the network architecture details of our multimodal sarcasm detection model, implemented in PyTorch (version 1.8.1) and running on a GeForce RTX 2070 GPU (8 GB memory). Text features are extracted using RoBERTa, trained extensively with longer steps, larger batches, more data, extended sequences, and dynamic masking. To ensure modality consistency, image features are obtained from the ViT in CLIP as the image encoder. Training is performed for 45 epochs with the Adam optimizer, using a learning rate of 1 × $10^{-5}$ and a batch size of 16. Complete model parameters are provided in Table 2.

Accuracy and weighted F1-score are employed as evaluation metrics, defined as follows:

(13) $$\begin{array}{cc} \; & Accuracy=\frac{\sum_{i}n_{ii}}{\sum_{i}\sum_{j}n_{ij}},\\ F1=\sum_{i} & (\frac{\sum_{j}n_{ij}}{\sum_{i}\sum_{j}n_{ij}}\times \frac{2\times Precision_{i}\times Recall_{i}}{Precision_{i}+Recall_{i}}), \end{array}$$

where $n_{ii}$ is the number of samples with true and predicted label *i*, and $n_{ij}$ is the number of samples with true label *i* and predicted label *j*. The weighted F1 metric is the weighted mean of all per-class F1 scores, with $Precision_{i}$ and $Recall_{i}$ representing the precision and recall for the *i*-th sentiment category:

(14) $$\begin{array}{cc} \; & Precision_{i}=\frac{n_{ii}}{\sum_{j}n_{ji}},\\ \; & Recall_{i}=\frac{n_{ii}}{\sum_{j}n_{ij}}, \end{array}$$

where $n_{ji}$ represents the number of samples with true label *j* and predicted label *i*.

### 4.3. Sensitivity and Robustness Analysis

To evaluate the stability of the proposed SGMLN, we investigated its sensitivity to decision thresholds and key training configurations. Since the model outputs probabilistic predictions optimized via binary cross-entropy loss, the decision boundary is implicitly determined by the learning objective rather than manually adjusted thresholds. In addition, the bidirectional KL-divergence constraint promotes consistency between modality predictions, improving probability calibration and stabilizing classification confidence. Consequently, the model performance remains remarkably stable even under moderate variations in decision thresholds.

This robustness is further evidenced by our evaluation of the model’s stability across a wide range of core hyperparameters, such as the learning rate and the KL-divergence loss weight. Experimental results indicate that our framework maintains consistent performance across a ±20% variation in these parameters. Such stability demonstrates that the SGMLN architecture is not overly dependent on specific parameter tuning, but rather benefits from its intrinsic design for robust affective alignment and cross-modal interaction. 

### 4.4. Baselines

Our proposed approach is systematically compared against multiple competitive baseline models to benchmark its effectiveness through detailed analysis.

(1) Unimodal. For unimodal sarcasm-detection tasks, we use the feature-extraction network for the respective modality as an encoder, followed by a fully connected layer for classification.

(2) HFM [23]. The Hierarchical Fusion Model (HFM) integrates textual and visual features in a stepwise manner, using attribute-level information to guide the fusion process.

(3) D&R Net [40]. The Decomposition and Relation Network (D&R Net) leverages semantic associations to isolate sarcastic cues, thereby improving prediction accuracy.

(4) HKE [12]. The Hierarchical Knowledge-Enhanced (HKE) model builds a layered architecture that incorporates external knowledge to better comprehend sarcastic expressions within context.

(5) CMGCN [41]. The Cross-Modal Graph Convolutional Network (CMGCN) models inter-modal inconsistencies by generating a graph-based representation for each modality, enabling cross-modal interaction learning.

(6) DCMG [35]. The Deep Cross-Modal Mapping Graph Convolutional Network (DCMG) constructs cross-modal correlation graphs from text–image pairs and applies graph convolution to capture inter-modal incongruous sentiment expressions.

(7) MILNet [31]. The Mutual-enhanced Incongruity Learning Network (MILNet) introduces both local and global incongruity modules guided by semantic signals, aiming to filter out irrelevant multimodal noise.

(8) DIP [16]. The Dual Incongruity Perceiving (DIP) Network focuses on identifying sarcastic elements by capturing semantic misalignments between images and corresponding text.

(9) FSICN [26]. The Fact-Sentiment Incongruity Combination Network (FSICN) captures sarcasm by jointly analyzing factual conflicts, emotional dissonance, and their fused representations across modalities.

(10) MuMu [42]. The Multimodal Mutual Learning (MuMu) Network enhances image–text alignment for sarcasm detection by leveraging pre-trained encoders for both visual and textual inputs, which are initialized from a large-scale contrastive language–image model.

(11) HIAN [27]. A hybrid interactive attention network (HIAN) uses text, images, and class words via interactive attention and transformer modules to enhance multimodal sarcasm detection.

(12) KnowleNet [21]. The Knowledge Fusion Network (KnowleNet) utilizes knowledge from the Concept library as prior knowledge and captures semantic similarity across modalities to detect sarcasm.

(13) DynRT [25]. The Dynamic Routing Transformer (DynRT) Network leverages a hierarchical co-attention mechanism to dynamically establish pathways for identifying inconsistencies across modalities in sarcasm detection.

### 4.5. Results and Analysis

As indicated in Table 3, SGMLN outperforms the advanced multimodal baseline, achieving a 4.13% increase in accuracy and a 4.83% increase in binary average F1 score. These improvements stem from a careful analysis of the baseline structure. Specifically, HFM and D&R Net represent initial efforts in multimodal sarcasm detection, using ResNet for visual features and LSTM for textual features. However, they underperform compared to baselines with more advanced language encoders such as BERT, as textual indicators are more relevant for identifying sarcasm than visual ones. In comparison, recent models—including CMGCN, HKE, MILNet, DIP, and KnowleNet—employ strategies to uncover subtle cross-modal inconsistencies, thereby enhancing sarcasm recognition. Yet, these models still lack fine-grained analysis of inconsistencies and cannot dynamically capture semantic relationships or sentiment interactions between text and images. SGMLN fills this gap by introducing sentimental knowledge, guiding cross-modal interaction between image features and the text modality using sentiment information in the text. Additionally, SGMLN implements a mutual learning module to facilitate knowledge transfer between the two modalities, thereby further enhancing the accuracy of multimodal sarcasm detection.

**Figure 4:**
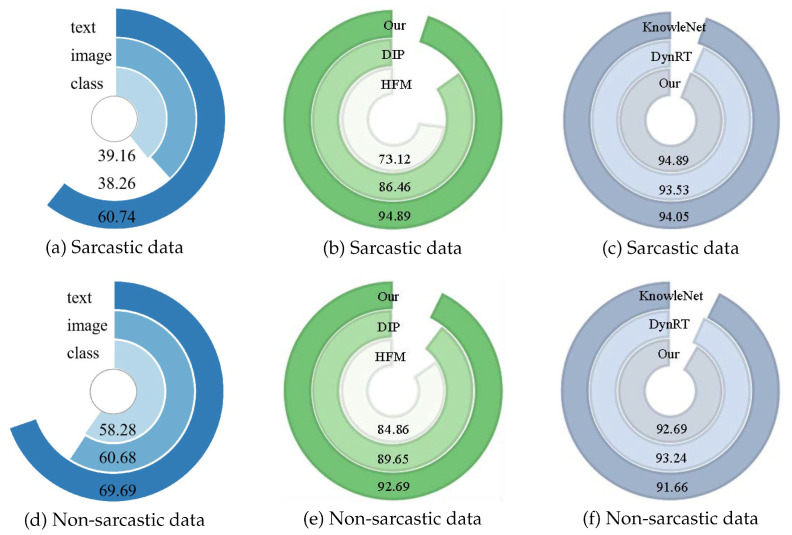
Recognition accuracy of each model on sarcastic and non-sarcastic data. Panels (**a**–**c**) correspond to sarcastic samples, while panels (**d**–**f**) correspond to non-sarcastic samples.

**Figure 5:**
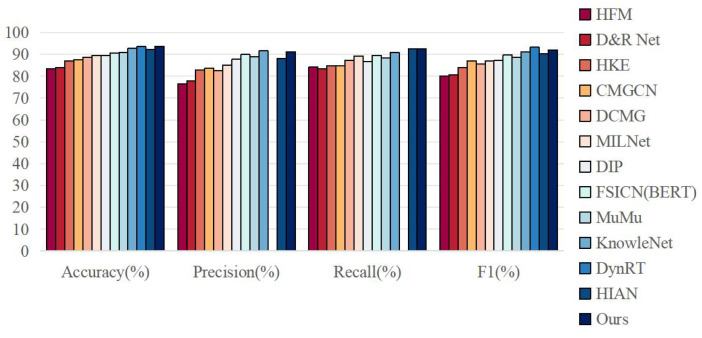
Visualization of experimental results.

Figure 4 shows that our model surpasses the DynRT model in sarcastic data recognition by a 1.36% accuracy gain, indicating that our approach is more effective at detecting sarcasm than DynRT. Additionally, our method achieves a 0.97% improvement over the KnowleNet model in identifying non-sarcastic samples, demonstrating greater robustness in distinguishing between sarcastic and non-sarcastic content. Finally, as shown in Figure 5, our method achieves higher accuracy and F1 scores than recent multimodal sarcasm detection models, underscoring its stronger ability to integrate features across modalities.

### 4.6. Ablation Studies

To assess the importance of each module in our multimodal sarcasm detection framework, we conduct ablation studies. Beginning with the full model, we remove individual modules to evaluate their contributions. Details for each model variant follow:

*w/o SentiGA*: In this variant, the Sentiment-Guided Attention (SentiGA) layer is removed to evaluate its contribution to cross-modal fusion. The model independently extracts textual and visual features using RoBERTa and the CLIP visual encoder, respectively. These features are then directly fed into the mutual learning framework for classification, without sentiment-guided alignment or fusion.

*w/o ML*: This variant removes the Mutual Learning (ML) module to assess its role in classifier collaboration. Instead of performing interactive knowledge exchange between classifiers, the sentiment-guided fused features are directly passed to a fully connected layer for final sarcasm classification.

Comparing these variants with the full SGMLN model enables a rigorous analysis of each component’s contribution. As shown in Table 4, removing the ML module (*w/o ML*) leads to a slight increase in the F1 score from 91.26% to 91.51%, but a noticeable decrease in overall accuracy from 93.63% to 93.16%. This observation indicates that the ML mechanism primarily improves prediction consistency and overall classification reliability by encouraging agreement between modality-specific classifiers. While this collaborative learning may introduce a minor precision–recall trade-off, it enhances the model’s global decision stability, which is reflected in the improved accuracy. These findings suggest that the ML module facilitates robust knowledge sharing rather than merely optimizing a single metric at the cost of noise.

Although the numerical differences between the full SGMLN and its simplified variants might appear relatively small, such improvements remain statistically and practically meaningful under high-accuracy conditions (i.e., above 90%). In this performance tier, gains are typically achieved by better handling ambiguous samples that involve subtle semantic–emotional inconsistencies between visual and textual cues. Therefore, our results suggest that the complete SGMLN architecture provides complementary cross-modal guidance rather than redundant complexity. By prioritizing prediction consistency and stability, the model achieves more reliable sarcasm detection across challenging real-world cases, justifying the inclusion of each proposed module.

Table 4 shows that all proposed components significantly improve sarcasm detection effectiveness. When the Sentiment-Guided Attention (SentiGA) module was removed, sarcasm detection accuracy decreased by 1.6%, validating the module’s effectiveness. We use a sentiment-guided attention layer to integrate sentiment knowledge into text and image features, enabling a more comprehensive multimodal understanding and facilitating the accurate identification of sarcasm via cross-modal inconsistencies. Removing the Mutual Learning (ML) module resulted in a 0.47% drop in accuracy, reinforcing its role in cross-modal knowledge transfer. The ML module enables features from each modality to capture complementary information, refining classification features and enhancing sarcasm detection performance.

### 4.7. Visualization

To evaluate the effectiveness of our model’s classification, we conducted confusion matrix analyses comparing the HFM baseline, unimodal, DIP, KnowleNet, DynRT, and our proposed models. As shown in Figure 4a,c,e, our model achieved a recognition accuracy on sarcastic data that was 0.84% higher than that of the top-performing multimodal model, DynRT, and 1.46% higher than that of the KnowleNet model, notably surpassing all others in sarcastic identification. This demonstrates that our model effectively detects inconsistencies between modalities. In addition, as shown in Figure 4b,d,f, our model also outperforms KnowleNet by 1.03% and DIP by 3.04% for non-sarcastic data recognition. We attribute this strong performance in recognizing non-sarcastic data to the integration of sentimental knowledge, which enhances the interaction and fusion of information between image and text modalities.

**Figure 6:**
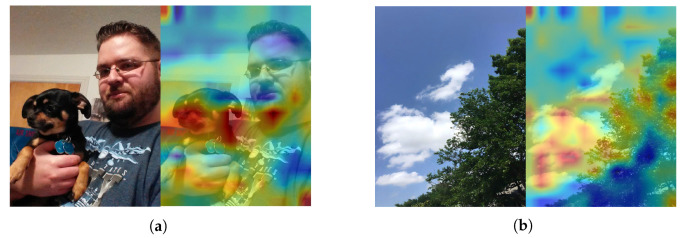
Visualization of two multimodal sarcasm examples. (**a**) As you can see, my dog Scout just loves having her picture taken. (**b**) This sure is a crazy storm we are getting.

To validate the efficacy of our proposed sentiment-guided attention layer, we visualized the attention distribution within it for two examples from the sarcasm dataset. Figure 6 displays the results. Incorporating sentimental knowledge and using the sentiment-guided attention layer can direct the focus to image regions with stronger sarcastic cues. For example, in Figure 6a, the attention primarily centers on the dog. This corresponds to the ‘dog’ concept in the text. This mutual reinforcement allows the model to correctly identify the example as sarcasm. In Figure 6b, the attention is mainly on the sky. This contradicts the ‘crazy storm’ concept in the text. As a result, this example is recognized as an instance of sarcasm. These observations confirm that the sentiment-guided attention layer effectively steers the network’s focus toward critical regions. This validates its effectiveness in sarcasm detection.

To further validate our model’s capabilities, we conducted a comparative analysis of predictive outcomes across eight cases. The analysis included the KnowleNet, DynRT, and SGMLN (ours) models. Comparative results are shown in Table 5. For non-sarcastic data instances such as (a), (b), (c), and (d), the KnowleNet model integrates external common-sense knowledge. However, it overlooks the importance of sentimental words in sarcasm detection. As a result, it misidentifies short sentences like (a) and (c). These sentences contain little external information but rich sentimental content. Likewise, the DynRT model does not account for sentimental information in the text. This limitation leads to wrong predictions for multimodal examples such as (a), (b), (c), and (d). The FSICN model integrates external emotional knowledge to find affective incongruities across modalities. Yet, it neglects the semantic alignment of multimodal features. This results in incorrect predictions for instances like (a) and (c) in multimodal examples. For sarcastic data, DynRT uses an iterative loop with an attention mechanism to refine categorical features. Still, its omission of sentiment information limits the accurate identification of multimodal cases with strong sentiment cues, such as (e), (g), and (h). In contrast, our model focuses on key sentimental words such as ‘happy’, ‘crazy’, ‘boring’, ‘fun’, ‘badly’, and ‘love’. It uses a sentiment-guided attention layer. This enables the effective extraction of sarcasm clues, leading to a better understanding of sarcasm and improved sarcasm detection performance.

## 5. Conclusions

This paper proposes SGMLN, a multimodal sarcasm detection framework that incorporates sentiment-guided attention to model the incongruity between visual and textual modalities. By treating sentiment as a guiding signal for cross-modal interaction and introducing a knowledge transfer mechanism based on a logistic distribution, the framework improves modality alignment and facilitates effective collaboration between image and text classifiers.

Experimental results on public datasets demonstrate consistently improved performance over existing multimodal sarcasm detection approaches. Ablation studies further clarify the role of each component: removing sentiment-guided attention weakens cross-modal alignment, while eliminating the mutual learning mechanism slightly increases F1 but reduces overall accuracy, indicating that collaborative learning primarily enhances prediction consistency and robustness. These findings suggest that the proposed modules make complementary rather than redundant contributions, particularly when handling ambiguous samples that involve subtle semantic–emotional inconsistencies.

From the perspective of the existing literature, our results extend those of prior studies on cross-modal interaction and affective modeling by showing that sentiment-aware fusion introduces affective guidance that enhances cross-modal alignment in sarcasm understanding. While recent multimodal large language models (MLLMs) exhibit strong general reasoning abilities, current evidence indicates that fine-grained sarcasm detection remains challenging due to subtle affective incongruity and high computational cost. In this context, SGMLN offers a complementary paradigm, showing that lightweight, task-specific architectures remain effective and practically deployable alongside large-scale pretrained models.

Overall, this work bridges affective knowledge modeling and collaborative multimodal learning, providing clearer insight into how sentiment-guided fusion contributes to sarcasm detection within the broader evolution of multimodal intelligence. Future work will focus on improving efficiency and exploring contrastive learning to further enhance robustness and generalization.

## Figures and Tables

**Table 1:** Statistics of dataset.

Statistics	Train	Test	Valid
All	19,816	2409	2410
Not Sarcasm	11,174	1450	1451
Sarcasm	8642	959	959

**Table 2:** Hyperparameters used in the training process.

Hyperparameter	Value	Description
batch_size	16	Batch size
lr	0.00001	Learning rate
epoch	45	Number of training epochs
weight_decay	$5\times 10^{-4}$	Weight decay coefficient

**Table 3:** Performance comparison of unimodal and multimodal approaches for multimodal sarcasm detection. The arrows (↑) indicate that higher values correspond to better performance. Bold values indicate the best performance in each column.

Modality	Method	Acc (%) ↑	Prec (%) ↑	Rec (%) ↑	F1 (%) ↑
Text	TextCNN [43]	80.03	74.29	76.39	75.32
	Bi-LSTM [44]	81.90	76.66	78.42	77.53
	SMSD [45]	80.90	76.46	75.18	75.82
	BERT [46]	83.85	78.72	82.27	80.22
	RoBERTa [47]	88.28	86.32	85.48	85.49
Image	ResNet [48]	64.76	54.41	70.80	61.53
	ViT [49]	67.83	57.93	70.07	63.43
Multimodal	HFM [23]	83.44	76.57	84.15	80.18
	D&R Net [40]	84.02	77.97	83.52	80.60
	HKE [12]	87.02	82.97	84.90	83.92
	CMGCN [41]	87.55	83.63	84.69	84.16
	DCMG [35]	88.65	82.69	87.21	85.73
	MILNet [31]	89.50	85.16	89.16	87.11
	DIP [16]	89.59	87.76	86.58	87.17
	FSICN(BERT) [26]	90.55	89.93	89.51	89.72
	MuMu [42]	90.73	88.81	88.44	88.62
	HIAN [27]	92.22	88.20	92.40	90.20
	KnowleNet [21]	92.69	**91.57**	90.85	91.21
	DynRT [25]	93.49	–	–	**93.21**
	**SGMLN (Ours)**	**93.63**	91.26	**92.63**	91.94

**Table 4:** Experimental results of ablation study.

Model	Acc (%)	F1 (%)	Pre (%)	Rec (%)
Ours	93.63	91.26	92.63	91.94
*w*/*o* SentiGA	92.03	89.89	89.51	90.28
*w*/*o* ML	93.16	91.51	86.90	93.90

**Table 5:** Representative examples of sarcasm and their predictions in the case study. Among them, ✓ indicates that the recognition is correct, and ✗ indicates that the recognition is incorrect.

	Image	Text	GT	KnowleNet	DynRT	FSICN	SGMLN
(a)	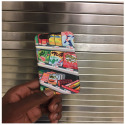	no regrets.	not sarcasm	✗	✗	✗	✓
(b)	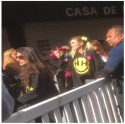	seria a lea michele com miley usando moletons da happy hippie?	not sarcasm	✓	✗	✓	✓
(c)	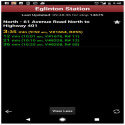	be beautiful.	not sarcasm	✗	✗	✗	✓
(d)	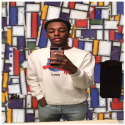	my bathroom is boring so since i’m extra i added some badly edited contemporary art	not sarcasm	✓	✗	✓	✓
(e)	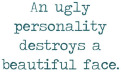	it’s so fun watching your bus become later and later …	sarcasm	✗	✗	✗	✓
(f)	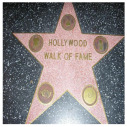	on a fb group and some asks “what is your favourite star sign?” who has a favourite? i posted this! i feel my may be lost on them.	sarcasm	✗	✓	✓	✓
(g)	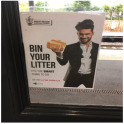	love your sexist stereotyping for this ad to promote binning litter.	sarcasm	✗	✗	✓	✓
(h)	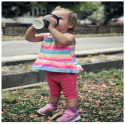	my niece loves coffee, just like her uncle.	sarcasm	✗	✗	✗	✓

## Data Availability

Publicly available datasets were analyzed in this study. The data were obtained from the Twitter multimodal sarcasm dataset introduced by Cai et al. (ACL 2019).
